# Elevation Shift in *Abies* Mill. (Pinaceae) of Subtropical and Temperate China and Vietnam—Corroborative Evidence from Cytoplasmic DNA and Ecological Niche Modeling

**DOI:** 10.3389/fpls.2017.00578

**Published:** 2017-04-18

**Authors:** Yi-Zhen Shao, Xian-Chun Zhang, Loc Ke Phan, Qiao-Ping Xiang

**Affiliations:** ^1^State Key Laboratory of Systematic and Evolutionary Botany, Institute of Botany, Chinese Academy of SciencesBeijing, China; ^2^Faculty of Biology, Vietnam National University of ScienceHanoi, Vietnam

**Keywords:** conservation, ecological niche modeling, endangered species, phylogeography, Quaternary

## Abstract

The “elevational shift” scenario has been proposed as a model to explain the response of cold-adapted organisms to Quaternary climatic fluctuations in Europe and North America. However, the elevational shift model has not been well-explored in eastern Asia, which is more topographically complex than the other Northern Hemisphere biogeographic regions. Here, we evaluated the role of elevational shift in the closely related firs, or *Abies* Mill., of subtropical and temperate China. These firs are typical alpine trees with sensitivity to climate change. We tested the elevational shift hypothesis in firs of China using phylogeographic methods and ecological niche models. Our phylogeographic analyses comprised mitochondrial and chloroplast polymorphisms surveyed across 479 individuals from 43 populations representing 11 species. M1 of the 11 mitotypes and C1 of the 25 chlorotypes were inferred as the ancestral haplotype, and they had the widest distribution. The results of our phylogeographic survey revealed multiple centers of genetic diversity in distinct geographic regions and no latitudinal trend. Moreover, our results showed range expansions for seven taxa during the last glacial (64.9–18.2 or 32.5–9.1 kya), and this was consistent with the Quaternary fossil record of *Abies* in China. Taken together, our findings support a historical biogeographic pattern in firs of glacial expansions, probably through corridors at lower elevation, and interglacial fragmentations, through isolation at higher elevation peaks. Therefore, *Abies* in China probably undergoes elevational shift in response to climate change. Facing the forecasting global warming, the risk of several critically endangered firs was further enhanced as these species would have little escape space *in situ* to higher altitudes. According to our ENMs, we proposed an *ex situ* conservation strategy in the southern Hengduan Mountains region of south western China.

## Introduction

Quaternary climate oscillations played an important role in population genetic structure and geographical distribution of species (Davis and Shaw, 2001). Depending on species' niche requirements, there are two common scenarios concerning the impact of Quaternary glacial-interglacial cycles on the spatial distribution of biodiversity (Hewitt, 2004). One is the “latitudinal shift” scenario for thermophilous, low elevation temperate species. Under this scenario, the historical interglacial periods facilitated range expansions from glacial refugia at low latitudes to higher latitudes (Schmitt and Rákosy, 2007; Andreia et al., 2016; Nicolas et al., 2017). Such latitudinal shifts led either to higher genetic diversity at lower latitudes than at higher ones (Hewitt, 1996) or to lower genetic diversity at lower latitudes than at higher ones (Petit et al., 2003; Lascoux et al., 2004; Cheddadi et al., 2009). The other inter-glacial cycle scenario is “elevational shift.” Under this scenario, cold-adapted organisms, such as alpine (or arctic) plants, responded to Quaternary climatic fluctuations by shifting along an altitudinal gradient (Galbreath et al., 2009; Awad et al., 2014; Bystriakova et al., 2014; Inoue and Berg, 2016). Such species not only migrated to lower latitudes but also to lower elevations, they had relatively wider distributions during glacial maxima but became restricted to higher mountains or sky islands during interglacial periods. Thus, elevational shifts lead to the actual pattern of multiple centers of genetic diversity, and to negligible trends in latitudinal diversity (Hewitt, 1996). Examples of species that experienced elevational shift include *Abies cilicica* (Awad et al., 2014) and *Asplenium fontanum* (Bystriakova et al., 2014).

The elevational and latitudinal scenarios have been previously documented in Europe (e.g., Bystriakova et al., 2014; Ruosch et al., 2016) and North America (e.g., Dechaine and Martin, 2004, 2005; Inoue and Berg, 2016). Nevertheless, few studies have sought to clarify elevational and latitudinal shifts in eastern Asia (Qiu et al., 2011; Liu et al., 2014). During the Quaternary glaciations, most regions in eastern Asia remained ice-free, with ice cover occurring as valley and piedmont glaciers and ice caps only in regions with high peaks (Shi et al., 1984, 1987; Rutter, 1995; Hewitt, 2000, 2004; Shi, 2002), which is in contrast to the extreme glaciation across Europe and North America. Latitudinal shifts have been detected in several warm-adapted species (e.g., Qiu et al., 2011; Liu et al., 2014), such as in *Juglans mandshurica* and *Euptelea pleiospermum* (Wang et al., 2016; Wei et al., 2016). However, few studies have been conducted on arctic-alpine species despite their potential importance for a comprehensive understanding of the impact of global warming on cold-adapted species.

Firs are the dominant trees in the boreal and temperate montane forests of the Northern Hemisphere (Cheng and Fu, 1978; Farjon, 2001). These plants have stringent biological requirements for humidity and temperature stability, which have made them good paleoclimatic indicators (Xu et al., 1980; Shi, 1996). The climatic requirements of firs are highly conserved phylogenetically (Xiang et al., 2000, 2004, 2009, 2015; Fan, 2006), and makes them a good model system to study historical elevational shifts as well as responses to ongoing climate changes.

The genus *Abies* is discontinuously distributed in the temperate and subtropical montane forests of the northern hemisphere, with subtropical and temperate China as the well-known center of diversity (Cheng and Fu, 1978; Farjon, 2001). Subtropical and temperate China is known for their complex topography which provides varied microhabitats for living organisms (Wu, 1980; Ying et al., 1993; Axelrod et al., 1996; Ying, 2001). There are 13–20 *Abie*s species in these adjacent regions with, an elevation from ca. 1,500 to 4,300 m (Cheng and Fu, 1978; Farjon, 2001; Rushforth, pers. comm.). Using morphological characters, *Abies* of China has been divided into as many as three questionably distinct subsections (Liu, 1971; Farjon and Rushforth, 1989), though all species form one clade according to recent molecular phylogenetic studies (Xiang et al., 2000, 2004, 2009, 2015; Shao and Xiang, 2015). Such a monophyletic group is suitable to study diversification processes by recovering intraspecific divergences and phylogeographic patterns for multiple-species (Liepelt et al., 2010; Liu et al., 2012). Thus, these *Abies* species provide an excellent system to investigate elevational shift through comparative phylogeography and ecological niche modeling (Ying, 2001; Qian et al., 2003; Liu et al., 2014).

In the present study, we conducted phylogeographic analyses and historical ecological niche prediction on Chinese subalpine and temperate firs, with the aims to: (i) evaluate the significance of the elevational shift scenario. If this were the case, we would expect to detect historical expansions during glacials and a resulting phylogeographic pattern. (ii) explore the genetic diversity structure and demographic history of the endangered firs with emphasis on the conservation under the conditions of ongoing climate change.

## Materials and methods

### Population sampling

We collected DNA materials for 475 individuals from 42 populations of Chinese subalpine and temperate firs, representing 10 species (Figure S1, Table S2). Four individuals of one population of *A*. *fansipanensis* QP Xiang, LK Fu, and Nan Li from Mt Fan Si Pan in northern Vietnam are included in the present study as a typical example of a highly endangered fir with only limited individuals (Xiang, 1997). Within each population, we sampled needles from eleven–twelve individuals growing at least 100 m apart except for six populations (pop. 2, 9, 14, 22, 36, and 43; Figure S1), where we could only sample four–eight individuals. We sampled two to 13 populations each of the following seven species: *A*. *chensiensis* Tiegh., *A*. *delavayi* Franch., *A*. *fabri* (Mast.) Craib, *A*. *forrestii* Coltm.-Rog., *A*. *georgei* Hand.-Mazz., *A*. *recurvata* Masters, and *A*. *squamata* Masters. Among them, 181 individuals from 17 populations (Nos. 1–13 and 33–36) of *A. chensiensis* and *A. recurvata* have been characterized for mtDNA and cpDNA sequence variation in a previous study (Shao and Xiang, 2015). To address the conservation implications of four other highly endangered fir species, we sampled only one population of each of them due to their limited availability and restrict geographic distributions: *A*. *fanjingshanensis* WL Huang, YL Tu, and SZ Fang (IUCN: endangered), *A*. *yuanbaoshanensis* YJ Lu and LK Fu (IUCN: endangered), *A*. *ziyuanensis* LK Fu and SL Mo (IUCN: endangered) and *A. fansipanensis* (IUCN: critically endangered; IUCN, 2016). Our sampling also included the following outgroup taxa: *A*. *bracteata* endemic to North America as outgroup in the mitotype analyses, and *A*. *alba, A*. *nordmanniana* (from Euro-Mediterranean), and *A*. *mariesii* from Japan as outgroup taxa in the chlorotype analyses. Our outgroup selection was based on a previous phylogenetic study (Xiang et al., 2015); only one individual was sampled for each outgroup. We present all sampling efforts in Table S2 and indicate samples that resulted from this study vs. a prior one comprising 181 individuals from 17 populations of *A. chensiensis* and *A. recurvata* (Nos. 1–13 and 33–36). Population sampling in the wild was strictly observed according to international rules (Nagoya, 2010).

### DNA extraction, PCR, and sequencing

We extracted total genomic DNA using a modified cetyltrimethyl ammonium bromide (CTAB) protocol (Rogers and Bendich, 1988) or DNA secure Plant Kit (Tiangen). We used the DNA extractions to isolate two mtDNA fragments, *nad*1 intron 2 and *nad*5 intron 4, and one cpDNA intergenic spacer, *trn*S/G, following procedures in Shao and Xiang (2015). We performed sequencing reactions using a DYEnamic ET Terminator Kit (Amersham Pharmacia Biotech), and detected labeled sequencing products on a MegaBACE 1000 (Amersham Biosciences, Buckinghamshire, UK). We deposited all newly generated sequences in GenBank under accession numbers KP635976–KP636041.

### Population structure and demographic analyses

We estimated molecular diversity indices, including the average pairwise differences per base pair between sequences (π) (Nei and Li, 1979), and the haplotype diversity (*h*) (Nei, 1987) for each taxon and population using DNASP version 4.10 (Rozas et al., 2003). Using *h* and π, we detected patterns of spatial genetic diversity using the GDivPAL function in Spads 1.0 (Dellicour and Mardulyn, 2014). We also calculated haplotype networks among mitotypes and chlorotypes, individually, in NETWORK version 4.2.0.1, with gaps treated as single events (Bandelt et al., 1999).

We used the program PERMUT (Pons and Petit, 1996) to calculate the average gene diversity within populations (*H*_S_), total gene diversity (*H*_T_) and two measures of population differentiation (*G*_ST_ and *N*_ST_). When *N*_ST_ estimates were significantly higher than the *G*_ST_-values, we inferred a phylogeographic structure. We performed an analysis of molecular variance (AMOVA) using the program ARLEQUIN 3.5 (Excoffier and Lischer, 2010) to determine the amount of genetic variation within and among the taxa and populations. In order to test the correlation between genetic and geographic distances, the mantel test was performed (Mantel, 1967; Nei and Li, 1979). We identified population groups that were geographically homogenous and maximally differentiated from each other using spatial analysis of molecular variance (SAMOVA; Dupanloup et al., 2002). We detected distinct populations (K) via a simulated annealing approach to maximize among population variance (*F*_CT_), and we chose the K between 2 and 10 where *F*_CT_ began to plateau.

We performed mismatch distribution analyses in the program ARLEQUIN 3.5 to detect historical population expansion events. When we detected an expansion event, we estimated its age using the formula *s* = 2 ut (Rogers and Harpending, 1992) where *t* is the age of the expansion, *u* = μ*kg* (μ is the substitution rate per nucleotide site per year, *k* is the average sequence length, and *g* is the generation time in years). In this study, we estimated expansions using a range of neutral substitution rates for cpDNA (μ = 2.61 × 10^−10^–4.02 × 10^−10^ ss^−1^ year^−1^) (Gernandt et al., 2008), an average haplotype sequence length (1,111 bp) in Pinaceae (Brown et al., 2004). In Pinaceae, the minimum estimate of generation time was 25 years (Brown et al., 2004). However, it should be mentioned that sexual maturity and effective generation time (when seedlings are actually recruited) of fir species in wild populations was also likely to be around 50 years (Cheng and Fu, 1978; Fan, 2006). Here, we calculated the historical expansion time under the two different minimum estimate of generation time (option 1: *g* = 25 years; option 2: *g* = 50 years).

### Ecological niche modeling

We performed ecological niche modeling (ENM) of Chinese subalpine and temperate firs in MAXENT (version 3.2.1; Phillips et al., 2006). The ENMS were not performed on species level for the extremely limited reliable occurrence records except for *A*. *chensinesis* (Fan, 2006; Shao and Xiang, 2015). For input into MAXENT, we obtained species occurrence data from the Chinese Virtual Herbarium (CVH, www.cvh.org.cn) and personal observations, including 112 records. These records involved 36 *A. chensiensis* records, 15 *A. delavayi* records, 11 *A. fabri* records, 8 *A. forrestii* records, 9 *A. georgei* records, 20 *A. recurvata* records, seven *A. squamata* records, 1 *A. fanjingshanensis* record, 1 *A. yuanbaoshanensis* record, 3 *A. ziyuanensis* records, and 1 *A. fansipanensis* record.

We used 19 BIOCLIM variables as environmental data for ENM representing five different time periods. Past and current data for these variables were available from the WorldClim database (www.worldclim.org). The data from WorldClim included baseline climate (BioClim layers for the period 1,950–2,000) at a spatial resolution of 30 arc s), data for the last glacial maximum (LGM; ~21,000 years BP) with spatial resolution of 2.5 arc min resolution simulated by CCSM model and MIROC model, and the last interglacial (LIG; ~120,000–140,000 years BP) period with spatial resolution in 30 arc s resolution. We obtained predictions for future climatic conditions in 2,050 and 2,080, from the International Center for Tropical Agriculture (www.ccafs-climate.org) based on the HadCM3. The HadCM3 model shows two scenarios (A2, and B2), which were represented in 2.5 arc min resolution Many climatic variables were highly correlated (|Pearson's *R*| ≥ 0.8). Therefore, our final ENMs were constructed using seven variables (mean diurnal range of temperature, temperature seasonality, max temperature of warmest month, mean temperature of driest quarter, mean temperature of coldest quarter, precipitation of driest, and warmest quarter) with limited covariance.

To construct ENMs, we conducted ten replicate runs with these parameters: default convergence threshold, maximum iterations (1,500), and 30% of the sites for model training (Waltari et al., 2007; Jezkova et al., 2009). We evaluated model performance using the area under the receiver operating characteristic curve (AUC) calculated by MAXENT.

### Pollen data collection

We initially surveyed more than 200 Quaternary fossil pollen records from both publications and database sources involving distinct species and time span, following Cao et al. (2013). During the last glaciation, *Abies* spp. occurred in 10 pollen records with reliable chronologies and sufficiently high sampling resolutions, covering the subtropical and temperate China (Liu and Ye, 1977; Zhou et al., 1978; Zhang et al., 1997; Sun and Li, 1999; Wu et al., 2002; Xu et al., 2002; Feng et al., 2007; Tang et al., 2007; Cook et al., 2011; see details in Table S1).

## Results

### Mitochondrial genealogy

**Figure 1:**
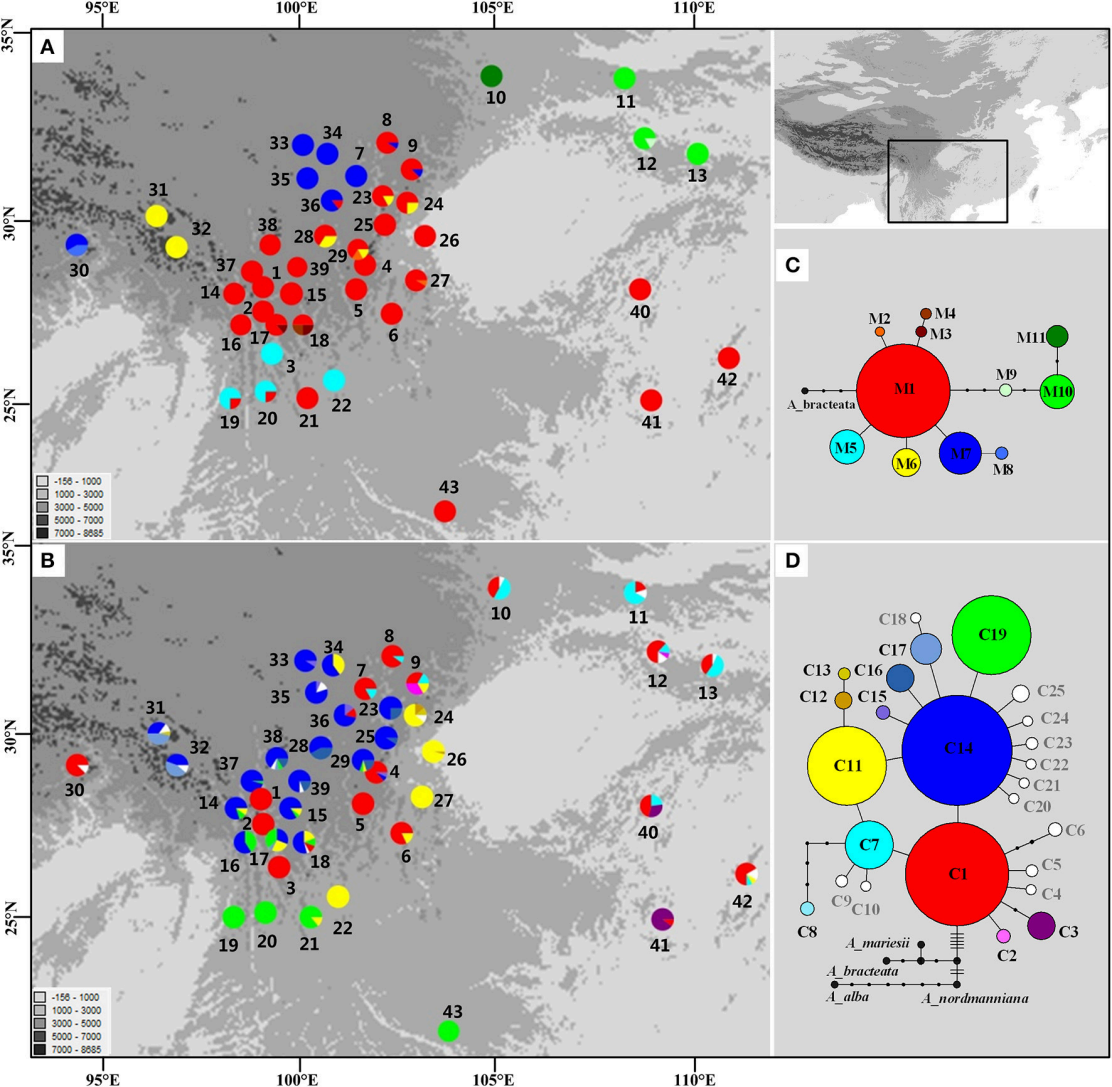
**Distributions and networks of mitotypes (A,C)** and chlorotypes **(B,D)** recorded in the Chinese subtropical and temperate firs. Private chlorotypes are shown in white. See Table S2 for population code numbers.

The length of the mtDNA fragments, *nad*1 intron 2 and *nad*5 intron 4, was conservative; 675 bp and 220 bp, respectively. The fragments contained seven point mutations and four indels resulting in 11 distinct mitotypes (M1–11; Table S3). Our results show that M1 has the widest distribution and is likely the ancestral haplotype according to the NETWORK analysis. M1 was the dominant haplotype in the central Hengduan Mountains region (Figure 1) and also occurred in Vietnam and southeast China which harbored four rare and critically endangered species, *A. fanjingshanensis, A. fansipanensis, A. yuanbaoshanensis*, and *A. ziyuanensis* (Pop. 40–43; Figure 1).

**Figure 2:**
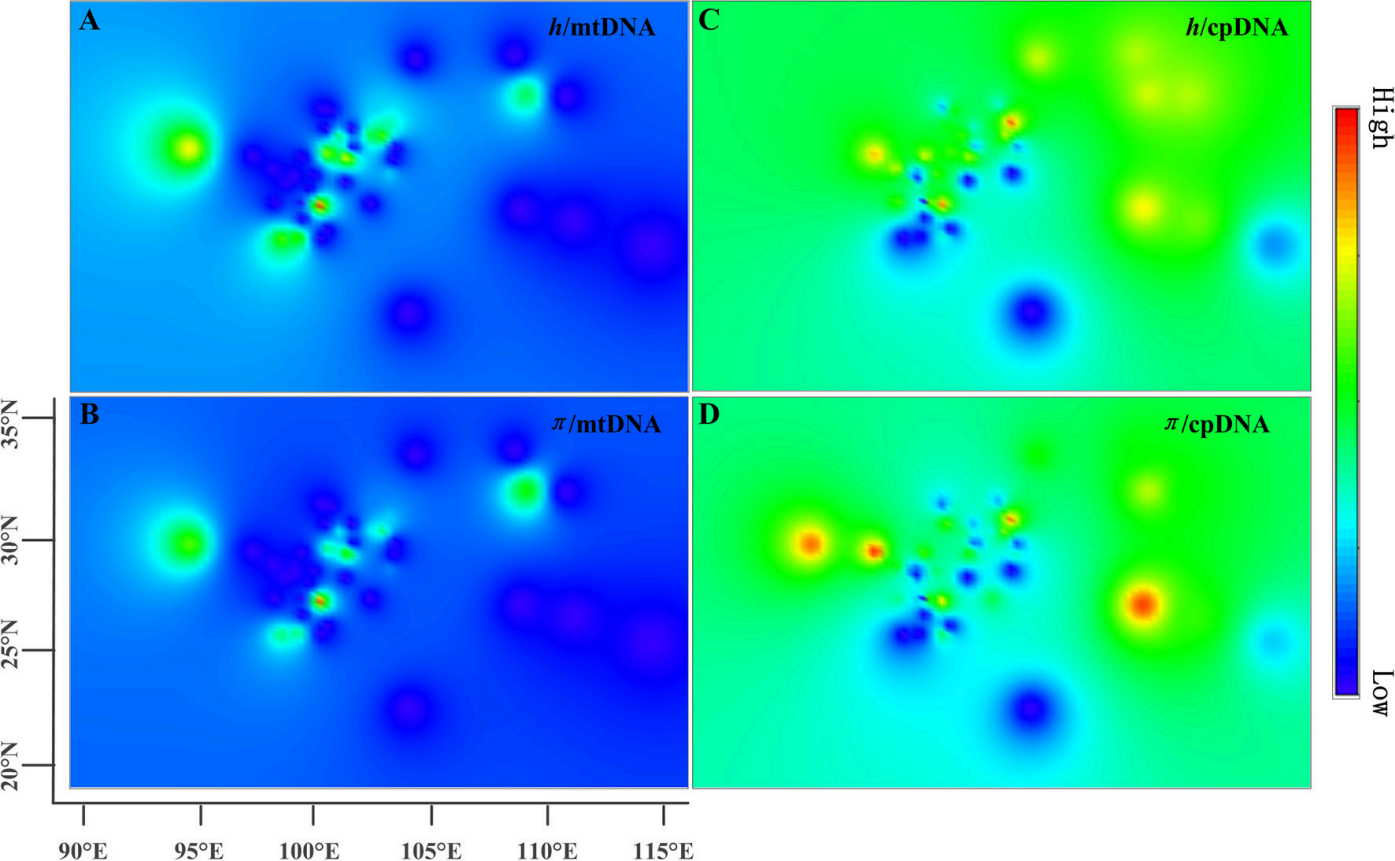
**Genetic diversity across the range of the Chinese subtropical and temperate firs**. The X-axis represents longitude and the Y-axis latitude. **(A)** pattern of mtDNA haplotype diversity (*h*); **(B)** pattern of mtDNA nucleotide diversity (π); **(C)** pattern of cpDNA haplotype diversity (*h*); **(D)** pattern of cpDNA nucleotide diversity (π).

The estimates of molecular diversity π and *h*, are summarized in Table S2. Diversity of mitochondria was high in several distinct geographic regions including the Hengduan Mountains, the Qinling-Daba Mountains, and Southeast China according to the results from Spads (green dots in Figures 2A,B). Within each species, the distribution of mitochondria diversity also didn't show any latitudinal trend (Table S2; Figures S4–S6). Our AMOVA analyses revealed that 12.36% of the total mtDNA sequence variation occurred among taxa, whereas 78.37% of the variation occurred among populations within taxa (Table 1). The mtDNA sequence variation showed *H*_S_ much lower than *H*_T_. Furthermore, mitotypes exhibited strongly isolated geographic distributions and a significant phylogeographic structure (*G*_ST_ < *N*_ST_; *P* < 0.05; Table 2). Genetic diversity was significantly correlated with geographic distances (*r* = 0.201, *p* = 0.037) according to our Mantel test (Table 2). However, no significant geographic division was revealed by the SAMOVA analyses.

**Table 1:** **Results of analyses of molecular variance (AMOVA) for species of ***Abies*** and DNA markers**.

**Species**	**Source of variation**	**mtDNA**			**cpDNA**		
		**df**	**Variation (%)**	**Fixation index**	**df**	**Variation (%)**	**Fixation index**
*A. chensiensis*	Among populations	12	97.52	F*_ST_* = 0.97520^**^	12	22.30	F*_ST_* = 0.22299^**^
	Within populations	131	2.48		131	77.70	
	Total	143			143		
*A. delavayi*	Among populations	8	61.34	F*_ST_* = 0.61338^**^	8	52.32	F*_ST_* = 0.52323^**^
	Within populations	90	38.66		90	47.68	
	Total	98			98		
*A. fabri*	Among populations	4	12.12	F*_ST_* = 0.12121^**^	4	34.19	F*_ST_* = 0.34186^**^
	Within populations	55	87.88		55	65.81	
	Total	59			59		
*A. forrestii*	Among populations	1	1.82	F*_ST_* = 0.01818	1	–1.09	F*_ST_* = –0.01091
	Within populations	22	98.18		22	101.09	
	Total	23			23		
*A. georgei*	Among populations	2	35.83	F*_ST_* = 0.35835^**^	2	22.56	F*_ST_* = 0.22562^**^
	Within populations	32	64.17		32	77.44	
	Total	34			34		
*A. recurvata*	Among populations	3	91.99	F*_ST_* = 0.91987^**^	3	27.24	F*_ST_* = 0.27239^**^
	Within populations	38	8.01		38	72.76	
	Total	41			41		
	Among populations	2	0.00	F*_ST_* = 0.00000^**^	2	–1.44	F*_ST_* = –0.01435
*A. squamata*	Within populations	33	0.00		33	101.44	
	Total	35			35		
In total	Among species	10	12.36	F*_CT_* = 0.12360	10	38.14	F*_CT_* = 0.38143^**^
	Among populations within species	32	78.37	F*_SC_* = 0.89421^**^	32	22.41	F*_SC_* = 0.36235^**^
	Within populations	436	9.27	F*_ST_* = 0.90728^**^	436	39.44	F*_ST_* = 0.60557^**^
	Total	478			478		

df, degrees of freedom;

** *P ≤ 0.001*.

**Table 2:** **Genetic diversity estimates and Mantel tests for species of ***Abies*** and DNA markers**.

**Species**		***H*_T_**	***H*_S_**	***G*_ST_**	***N*_ST_**	**Mantel test (*r*)**
*A. chensiensis*	mtDNA	0.710 (0.108)	0.055 (0.030)	0.922 (0.045)	0.969 (0.019)^*^	*r* = 0.525, *p* = 0.001
	cpDNA	0.488 (0.102)	0.369 (0.087)	0.245 (0.035)	0.220 (0.033)^*^	*r* = 0.249, *p* = 0.105
*A. delavayi*	mtDNA	0.528 (0.109)	0.185 (0.085)	0.650 (0.130)	0.655 (0.1055)^*^	*r* = 0.254, *p* = 0.115
	cpDNA	0.700 (0.058)	0.365 (0.104)	0.479 (0.155)	0.508 (0.173)^*^	*r* = 0.490, *p* = 0.006
*A. fabri*	mtDNA	NC	NC	NC	NC	*r* = –0.542, *p* = 0.985
	cpDNA	NC	NC	NC	NC	*r* = 0.627, *p* = 0.040
*A. forrestii*	mtDNA	NC	NC	NC	NC	NC
	cpDNA	NC	NC	NC	NC	NC
*A. georgei*	mtDNA	NC	NC	NC	NC	*r* = 0.923, *p* = 0.327
	cpDNA	0.886 (0.028)	0.596 (0.098)	0.328 (0.222)	0.491 (0.157)	*r* = 0.979, *p* = 0.174
*A. recurvata*	mtDNA	0.524 (0.206)	0.071 (0.071)	0.864 (0.178)	0.864 (0.178)	*r* = –0.375, *p* = 1.000
	cpDNA	NC	NC	NC	NC	*r* = 0.660, *p* = 0.100
*A. squamata*	mtDNA	NC	NC	NC	NC	NC
	cpDNA	0.472 (0.126)	0.455 (0.149)	0.037 (0.177)	0.031 (0.199)^*^	*r* = 0.028, *p* = 0.513
In total	mtDNA	0.588 (0.070)	0.119 (0.030)	0.798 (0.051)	0.901 (0.033)^*^	*r* = 0.201, *p* = 0.037
	cpDNA	0.827 (0.019)	0.387 (0.041)	0.532 (0.049)	0.597 (0.047)^*^	*r* = 0.227, *p* = 0.010

H_S_, average genetic diversity within populations; H_T_, total gene diversity; G_ST_, interpopulation haplotype differentiation; N_ST_, interpopulation haplotype differentiation taking into account sequence difference;

* *, N_ST_ is significantly different from G_ST_ (P < 0.05); NC, not computed due to small sample size or low variation among populations*.

### Chloroplast genealogy

In the chloroplast DNA data, we detected six indels and 17 substitutions resulting in 25 chlorotypes (C1–25; Table S4). Five chlorotypes had frequencies above 5% (C1, C7, C11, C14, and C19). Fifteen chlorotypes (C2, C4–6, C8–10, C12, C18, and C20–25) were fixed in single species, while the remaining ten were shared among species (Figure 1B). The chlorotype C1 possessed the widest geographic distribution and may be ancestral haplotype according to the NETWORK analysis (Figure 1).

The cpDNA exhibited higher genetic diversity than the mtDNA according to the Spads analysis (Figure 2; cpDNA in green). The cpDNA possessed multiple centers of diversity according to the indices *h* and π (Figures 2C,D; yellow and red dots). Within each species, the distribution of cpDNA diversity also didn't show any latitudinal trend (Table S2; Figures S4–S6). Of the variation among chlorotypes, 38.14% was among taxa and and 39.44% was within populations (Table 1). Chlorotypes exhibited significant phylogeographic structure (*G*_ST_ < *N*_ST_; *P* < 0.05), and *H*_S_ was significantly lower than *H*_T_ (Table 2). However, we did not detect a significant division among populations according to SAMOVA analysis, but the Mantel test revealed a significant correlation between genetic and geographic distances (*r* = 0.227, *P* = 0.010; Table 2).

### Population demographic history

We performed mismatch distribution analyses on Chinese subalpine and temperate firs taken together and each species individually using cpDNA sequence data. We analyzed seven species for mismatched distributions (*A. chensiensis, A. delavayi, A. fabri, A. forrestii, A. georgei, A. recurvate*, and *A. squamata*) and excluded the four rare and endangered species (*A. fanjingshanensis, A. fansipanensis, A. yuanbaoshanensis*, and *A. ziyuanensis*) because of the small sample size (No. pop = 1).

**Figure 3:**
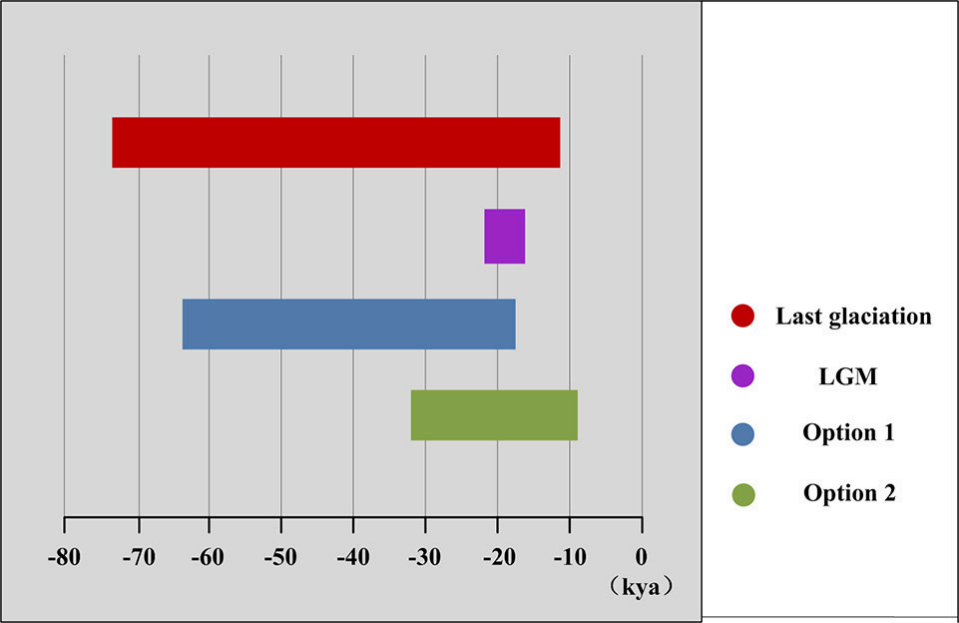
**Duration of demographic expansions detected by mismatch distribution analyses**.

The mismatch distribution results rejected the demographic expansion hypothesis for the Chinese subalpine and temperate firs as a whole (*P*_SSD_ = 0.000; Figure S2). However, the expansion hypothesis was supported for all of the seven species analyzed separately except for *A. delavayi* (*P*_SSD_ = 0.078; Figure S2). We determined that the demographic expansion of the six species probably occurred in 64.9–18.2 kya (option 1: *g* = 25 years) or 32.5–9.1 kya (option 2: *g* = 50 years; Figure 3, Table 3).

**Table 3:** **Mismatch distribution analysis for chloroplast DNA sequence data**.

**Species**	**SSD (*P-*value)**	**RAG (*P*-value)**	**τ**	**t*_min_***	**t*_max_***
*A. chensiensis*	0.002 (0.271)	0.129 (0.489)	0.537 (0.324–0.805)	24.052 (14.512–36.046)	37.042 (22.349–55.528)
*A. delavayi*	0.143 (0.078)	0.209 (0.060)	NC	NC	NC
*A. fabri*	0.015 (0.053)	0.138 (0.077)	0.941 (0.631–1.379)	42.936 (28.791–62.921)	64.910 (43.526–95.123)
*A. forrestii*	0.028 (0.090)	0.188 (0.130)	0.764 (0.232–1.385)	34.860 (10.586–63.240)	52.701 (16.003–95.606)
*A. georgei*	0.015 (0.560)	0.047 (0.630)	0.398 (0.000–4.146)	18.160 (0.000–189.2)	27.454 (0.000–285.991)
*A. recurvata*	0.008 (0.229)	0.122 (0.255)	0.754 (0.367–1.303)	33.771 (16.438–58.361)	52.010 (25.315–89.880)
*A. squamata*	0.017 (0.130)	0.147 (0.440)	0.445 (0.039–1.270)	20.30 (1.780–57.948)	30.696 (2.690–87.605)

*τ, time in number of generations elapsed since the sudden expansion episode; t, absolute time in kya; 95% CI estimated by Arlequin for τ and the corresponding timescales are indicated in parentheses; SSD, sum of squared deviations; RAG, the Harpending's Raggedness index. NC, not computed due to historical expansion was not suggested*.

### Ecological niche modeling

**Figure 4:**
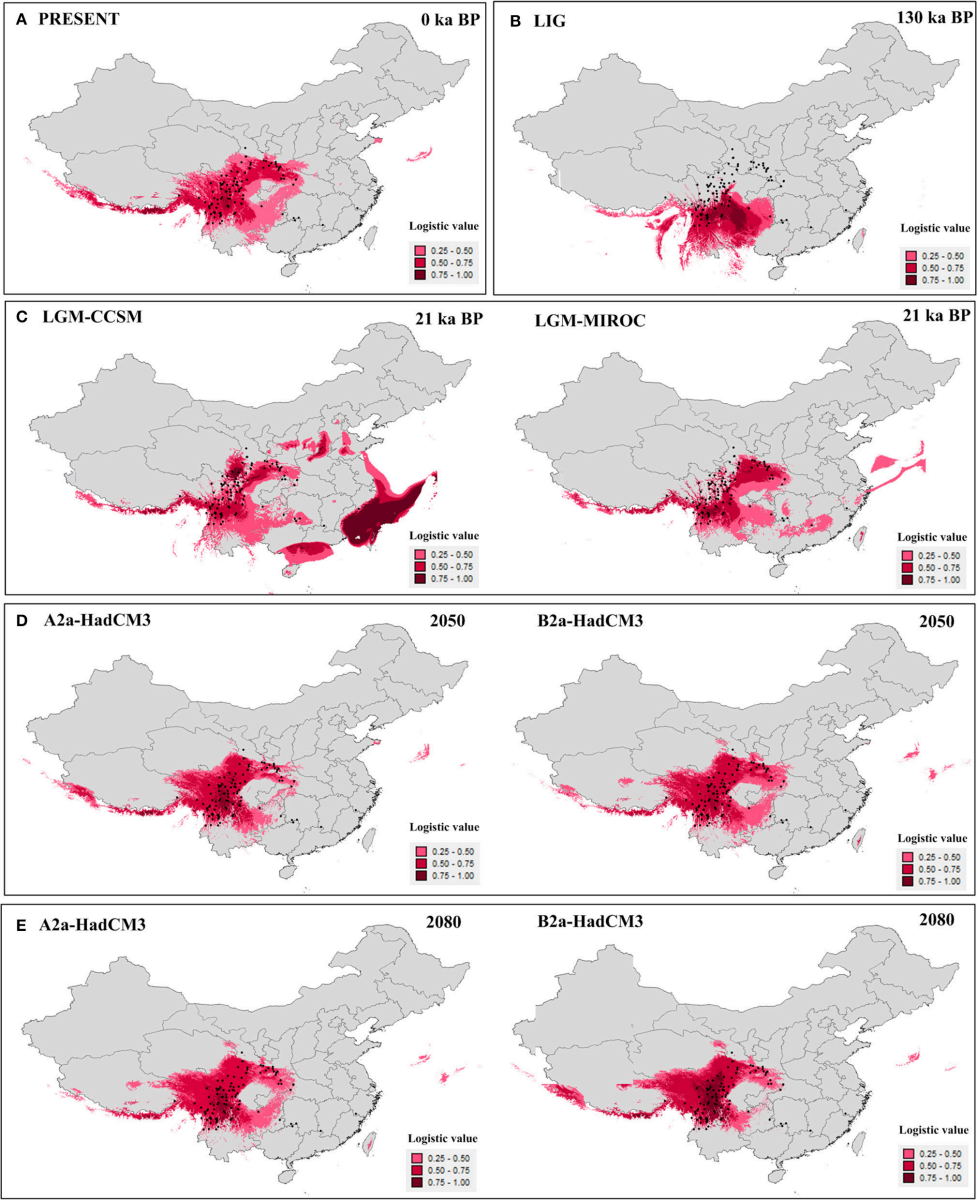
**Results of ecological niche modeling of Chinese subtropical and temperate firs. (A)** Predicted distribution probability (in logistic value) for current climatic conditions. **(B)** Average projection of the model to the last interglacial (c. 120–140 kyr BP). **(C)** Average projections of the model to the last glacial maximum [c. 21 kyr before present (BP)] using climatic variables under the Community Climate System Model (CCSM) and Model for Interdisciplinary Research on Climate (MIROC). **(D,E)** Average projections of the model to the future [2,050 **(D)** and 2,080 **(E)**] using climatic variables under the HadCM3 model according to two distinct scenarios (A2 and B2). Black dots present extant occurrence points.

The value of the AUC test (mean ± *SD*) for the ecological niche modeling, averaged across all 10 runs, was very high (0.96 ± 0.005). Models for the present (Figure 4A) and last interglacial (Figure 4B) showed contracted ranges compared to the LGM (Figure 4C), indicating losing of suitable habitat in south, central, and east China. The future predictions of suitable habitat were narrower than for the present day, and ranges were further reduced between 2,050 (Figure 4D) and 2,080 (Figure 4E). Except the Models for the LGM, the suitable habitats of the four highly endangered firs in southeast China were always absent (Figure 4). As for other seven species, the suitable habitats were slightly changed in all time periods bun not in the LIG. During the LIG when globe warming, suitable habitat of fir species distributed in northern part of the Hengduan Mountains region was contracted heavily, such as *A*. *recurvata, A*. *fabri, A*. *forrestii*, and *A*. *squamata* (Figure 4, Figure S1).

### Pollen-based distribution during the last glaciation

Palynological data indicated that *Abies* were much more common in lowland forests of central (no. 2,3,8) and eastern (no. 1,4,5,7,9) China during the last glaciation (Figure S3). This pattern depicted wider distribution during the last glaciation than today, Except for three pollen records from northeastern China (no. 1, 4, and 8), the pollen-based distribution pattern was highly consistent with the ENMs for the LGM by CCSM model (Figure 4C).

## Discussion

### The elevation shift of the chinese subtropical and temperate firs during the quaternary

Previous studies concerning arctic-alpine species from Europe and North America such as *Sedum lanceolatum* (Dechaine and Martin, 2005), *Asplenium fontanum* (Bystriakova et al., 2014), and *Abies cilicica* (Awad et al., 2014), indicate glacial expansion of elevational shift scenario. This “elevation shift” scenario had not been well-documented in arctic-alpine species of East Asia, and places constraints on its application to biodiversity conservation. By combining molecular phylogeography and ENM, we explored how the Chinese subtropical and temperate firs (typical alpine species) in eastern Asia responded to Quaternary climate change.

Two distinct expansion models have been proposed by Hewitt (1996) to distinguish elevational shift from latitudinal shift. The pioneer expansion model of latitudinal shift is characterized by decrease of genetic diversity along latitudes during interglacial expansion (Hewitt, 2004; Yan et al., 2012; Andreia et al., 2016; Nicolas et al., 2017). The phalanx expansion model of elevational shift supposes glacial expansions without significant reduction of genetic diversity along latitudes, resulting in multiple centers of diversity but without decreased genetic diversity along latitude (Galbreath et al., 2009; Awad et al., 2014; Bystriakova et al., 2014; Inoue and Berg, 2016). Based on current phylogeographical patterns, the phalanx expansion model via elevational shift was potentially active in Chinese subtropical and temperate firs. Firstly, multiple centers of fir diversity were discovered in the Hengduan Mountains region, Qinling-Daba Mountains region and in Southeast China, indicating no latitudinal decreasing trends (Figure 2). A similar pattern was also found within each species (Table S2; Figures S4–S6). Secondly, relatively low genetic diversity of mtDNA and cpDNA were found both within populations and species across Chinese subalpine and temperate firs (Table S2). Such low genetic diversity or strong homogeneity could be the result of rapid recolonization following severe bottleneck effects, accompanied by climate-driven elevation shifts along a local elevation gradient (Hewitt, 2000; Jaramillo-Correa et al., 2004; Jiang et al., 2011). This result is consistent with similar processes reported in the Mediterranean region and North America (Ziegenhagen et al., 2005; Jaramillo-Correa et al., 2008; Wang et al., 2011). In addition, the population diversity structure of *Abies* in subtropical and temperate China is similar to that of congeners in North America and Europe especially by having differentiation among populations (mtDNA: 90.73%; cpDNA: 60.56%; Table 1) significantly greater than the averages of conifers (mtDNA: 76.4%; cpDNA: 16.5%; reviewed in Petit et al., 2005) (Liepelt et al., 2002; Fady-welterlen, 2005; Jaramillo-Correa et al., 2008; Fady and Conord, 2010; Wang et al., 2011). Greater differentiation of conifers among populations may result from recent habitat fragmentations or severe bottleneck effects due to global warming since the Holocene epoch (Hewitt, 2004; Jaramillo-Correa et al., 2004; Jiang et al., 2011). Thus, the phylogeographical pattern of Chinese subtropical and temperate firs could be considered potential evidence of elevational shift, in general, and of the phalanx model in particular.

More directly evidences were further revealed by the expansion time estimation and ecological niche modeling. We estimated that the ranges of most firs (i.e., taken individually, except *A. delavayi*) expanded during approximately 64.9–18.2 kya (option 1: *g* = 25 years) or 32.5–9.1 kya (option 2: *g* = 50 years; Table 3; Figure S2). These two timing of the expansion were highly coincident with the last glaciation in China, which was 73–10.4 kya with a maximum during 21–18 kya (Yi et al., 2004, 2005, 2007; Zhao et al., 2011). Specifically, range expansion of *Abies* in subtropical and temperate China might have commenced shortly after the beginning of the last glacial cycle and continued throughout the last glacial maximum (option 1) or since the middle of the last glaciation till the end (option 2; Figure 3). Both the two options indicated the glacial expansions via elevational shift.

Curiously, we did not detect evidence for recent, historical range expansion in all subtropical and temperate China and Vietnam firs considered together or in *A. delavayi*. For *A. delavayi*, distribution fragmentation could be easily inferred by its typical high-altitude distribution, extending from the southern part of Hengduan Mountains region to north Vietnam. For the whole Chinese subtropical and temperate firs, multiple asynchronous expansions, multiple independent refugia *in situ* and complex topography in this region might be responsible for the undetectable in mismatch distribution analyses (Cun and Wang, 2010; Yan et al., 2012; Qin et al., 2013). Such analyses seemed to underestimate the possibility of historical expansions in some situations. Nevertheless, our historical distributional projections showed that firs of subtropical and temperate China and Vietnam as a whole underwent an expansion from the LIG to the LGM; that is, during a period of global cooling. Distributional projections for the present and future show ongoing range contractions (Figure 4) coincident with continued global warming. Although such analysis was not conducted at the species level due to insufficient occurrence records except for *A. chensiensis*, we believed that this tendency should be well-represented in respective taxa because of their conservative ecological niche requirement (Cheng and Fu, 1978; Farjon, 2001; Fan, 2006). The accuracy of ENMs was further validated by the high similarity between the LGM-CCSM prediction (Figure 4C) and pollen-based distribution during the last glaciation (Figure S3). Two prior studies modeled future geographic distributions of six fir species of China, Japan, and Pakistan, and determined that those species would all lose their suitable habitat in the lowlands region in response to ongoing global warming (Tanaka et al., 2012; Ali et al., 2014).

Many plant species have strong preferences for a narrow range of climatic conditions. Therefore, past climatic conditions can be inferred from the palynological records of plants (Tinner and Lotter, 2006). Palynological data indicate that *Abies* were much more common in lowland forests of central and eastern China during the last glaciation (Figure S3) when the mean annual temperature was 5–10°C lower than today (Xu et al., 1980; Shi, 1996; Cao et al., 2013). Moreover, the abundance of *Abies* pollen in central China decreased with the onset of the interglacial period, which was warmer and less humid than the glacial one. The current mountain top distribution of several highly endangered fir species (e.g., *A. beshanzuensis, A. fanjingshanensis, A. yuanbaoshanensis*, and *A. ziyuanensis*) is living evidence of interglacial fragmentation (Cheng and Fu, 1978; Xiang, 2001; Jiang et al., 2015). Even though deforestation in China had been significant in past decades, these fir species were not a main target due both to their distribution along mountain tops or along the tree line away from human settlements, and to the low-quality wood properties (Fan, 2006). In summary, our results including genetic diversity, mismatch distribution analyses, ecological niche models, combining evidence from other lines involving fossil records and the current distribution patterns, led to a conclusion that the Chinese subtropical and temperate firs experienced elevational shift during the Quaternary climate change.

### Implications for the conservation of the endangered firs

Our results show that Chinese subtropical and temperate firs have undergone major habitat fragmentation and genetic isolation and differentiation since the Holocene. The main goal of biological conservation is to maintain as many of the important genetic building blocks of the species as possible so that the evolutionary progress is not constrained (Fraser and Bernatchez, 2001; D'Amen et al., 2013). Thus, defining conservation units with evolutionary differences is necessary for these economically and ecologically valuable threatened firs (e.g., de Querioz, 1998; Fraser and Bernatchez, 2001).

In subtropical and temperate China and Vietnam, four species of *Abies* are listed as critically endangered due to their restricted distribution on isolated mountain tops and small population sizes: *A*. *beshanzuensis, A. fanjingshanensis* (Pop. 40), *A. yuanbaoshanensis* (Pop. 41) and *A. ziyuanensis* (Pop. 42) in warm, temperate China and *A. fansipanensis* (Pop. 43) in Vietnam (Fu, 1992; Xiang, 2001). *Abies beshanzuensis* was not included in this study because its population comprises only three individuals and is too ecologically fragile for sampling. The other four species all possessed the M1 ancestral mitotype but had high cpDNA diversity (Figure 1). This observation implied a possible shared ancestral gene pool which was fragmented during interglacials (Jaramillo-Correa et al., 2006; Loehr et al., 2006; Dubreuil et al., 2010). Specifically, the total number of cpDNA haplotypes was three, two, four, and one in *A. fanjingshanensis* (C1, C3, C7), *A. yuanbaoshanensis* (C1, C3), *A. ziyuanensis* (C1, C5, C7, C11), and *A. fansipanensis* (C19), respectively (Figure 1). These distinct chloroplast haplotypes in each species highlight the distinct evolutionary histories of the species (Liepelt et al., 2009; Jay et al., 2012), thus each may represent separate conservation units.

For the species sensitive to environmental change, conservation planning must take into consideration the climatic envelope of the species and its present geographic distribution (Root et al., 2003; Thomas et al., 2004; Forest et al., 2007). The climatic envelope of *Abies* has been shrinking in geographic space since the last ice age and will probably continue to do so under present global warming conditions (Figure 4). The reasons are various, such as pest and disease damage, human disturbance, and habitat destruction. Unfortunately, *Abies* may have limited opportunity to escape global warming through elevational shift, because most species already occur at or nearly at the maximum available altitude: For example, *A. beshanzuenesis* occurs on Baishanzu Mountain (1,857 m) at 1,750 m, *A. fanjingshanensis* is restricted to 2,050–2,390 m on Fanjing Mountain (2,494 m), *A. yuanbaoshanensis* is distributed on Yuanbao Mountain (2,081 m) at 1,900–2,000 m, and *A. ziyuanensis* is located on Shunhuang Mountain (1,882 m) at 1,700 m (Fan, 2006; Li et al., 2012). While some species of *Abies* can move upwards, they may not be able to do so at a fast enough rate (Huck et al., 2012; Moritz and Agudo, 2013). Based on the meta-analyses, Chen et al. (2011) proposed that the distributions of many terrestrial organisms currently shifted to higher elevations at a median rate of 11.0 m per decade or to higher latitudes at a median rate of 16.9 km per decade to accomodate global warming. Such a high rate is difficult to achieve for conifers, which have a two (or longer) year reproductive cycle (Liu, 1971; Farjon and Rushforth, 1989; Xiang, 2001).

*In situ* conservation is the current govermentnal strategy for these *Abies* species, but it is clearly not a permanent solution. In fact, there is not any suitable habitat for these highly endangered firs in actual or 2,050/2,080 models (Figure 4). Moreover, a massive suitable habitat contraction occurred in the northern part of the Hengduan Mountains region during the LIG (Figure 4B). Facing the forecasted global warming, the suitable habitats of *A*. *recurvata, A*. *fabri, A*. *forrestii*, and *A*. *squamata* were believed to be heavily contracted (Figure 4B, Figure S1). Thus, we propose that these species urgently require *ex situ* management in botanical gardens in areas of suitable climate with dedicated breeding programs. According to our ENMs (Figure 4), the suitable habitats for breeding firs were stable in the southern Hengduan Mountains region during each time periods. At present, more than 30 National Nature Reserves are located in the Hengduan Mountains, and also some relevant state-grade research institutes, such as the Kunming Institute of Botany, Chinese Academy of Sciences (Chen et al., 2009). These specific gardens and their existing programs would strengthen the development of *ex situ* conservation.

## Data accessibility

The matrix of plastid DNA alignments is included as Tables S3, S4 of the Supporting Information, respectively. Original sequences of the three plastid regions are available in the GenBank database (Accession nos. KP635976–KP636041), details see Table S5.

## Author contributions

Conceived and designed the experiments: QX. Performed the experiments and analyzed the data: YS. Contributed reagents/materials/analysis tools: XZ and LP. Wrote the paper: YS and QX.

### Conflict of interest statement

The authors declare that the research was conducted in the absence of any commercial or financial relationships that could be construed as a potential conflict of interest.

## Supplementary material

Figure S1 **Sampling locations of 43 populations of Chinese subtropical and temperate firs**. Each taxon is represented by distinct color and shape.

Figure S2 **The mismatch distributions for seven fir species in subtropical and temperate China based on cpDNA**. *A. chensiensis, A. delavayi, A. fabri, A. forrestii, A. georgei, A. recurvata, A. squamata*, and the Chinese subtropical and temperate firs as a whole are presented in **(A–H)**, respectively.

Figure S3 **The extant distribution of *Abies* in China and 10 pollen records during the last glaciation**. The red shows the present geographical ranges and the black triangle indicates the distribution of 10 pollen records in the subtropical and temperate China.

Figure S4 **Distributions and networks of mitotypes and chlorotypes recorded in *Abies chensiensis***. Private chlorotypes are shown in white. See Table S2 for population code numbers.

Figure S5 **Distributions and networks of mitotypes and chlorotypes recorded in *Abies delavayi***. Private chlorotypes are shown in white. See Table S2 for population code numbers.

Figure S6 **Distributions and networks of mitotypes and chlorotypes recorded in *Abies fabri***. Private chlorotypes are shown in white. See Table S2 for population code numbers.

Table S1 **Ten fossil records of Abies spp. in eastern and central China during the last glaciation**.

Table S2 **Details of sample locations and sample sizes of 43 populations and outgroups**. For each population, estimates of haplotype diversity (h), nucleotide diversity (n) and frequencies of haplotypes for mitochondrial DNA and chloroplast DNA sequences are also indicated.YN, Yunnan; SC, Sichuan; GS, Gansu; SX, Shanxi; CQ, Chongqing; HB, Hubei; GZ, Guizhou; HN, Hunan; GX, Guangxi; N, samples size. ^*^, highly endangered species. #, outgroups.

Table S3 **Variable sites of the 11 mitotypes detected in Chinese subalpine and temperate firs**.

Table S4 **Variable sites of the 25 chlorotypes detected in Chinese subalpine and temperate firs**. #, AAGA; ^*^, ACGGA; &, TATCTA; α, ATTATTG; β, CTATTGTAAAGATATTAGTTGCTCAAGG; γ, ACTAC.

Table S5 **Species names and GenBank accession numbers of DNA sequences used in this study**. ^*^ means newly generated sequences.
