# Supplementary figures and images for: Elevation Shift in Abies Mill. (Pinaceae) of Subtropical and Temperate China and Vietnam—Corroborative Evidence from Cytoplasmic DNA and Ecological Niche Modeling
Source: Front Plant Sci. 2017 Apr 18;8:578. doi: 10.3389/fpls.2017.00578 (PMC5394127; doi:10.3389/fpls.2017.00578)

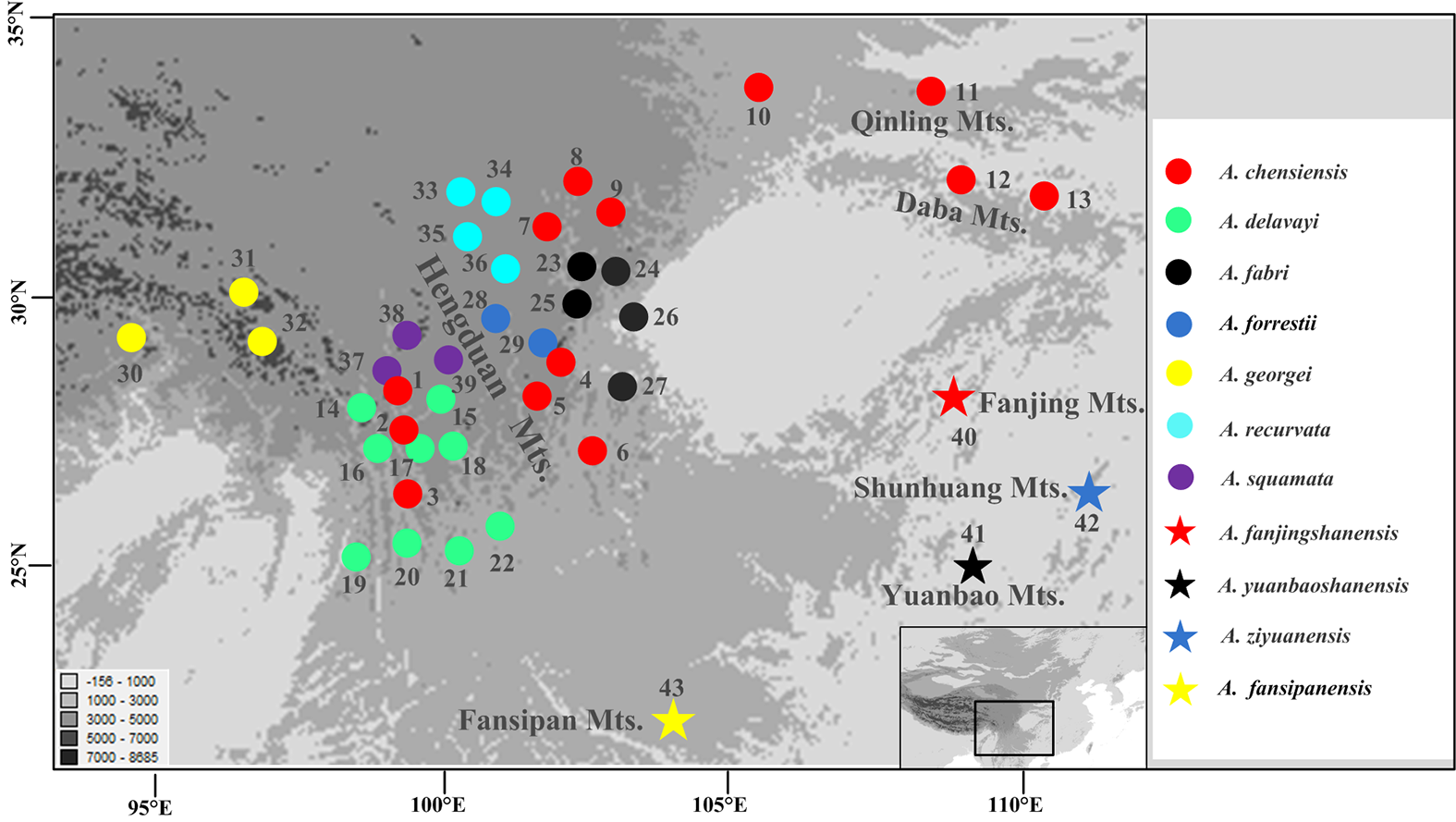

Supplement: Figure S1 — Sampling locations of 43 populations of Chinese subtropical and temperate firs. Each taxon is represented by distinct color and shape. [file Image1.TIF]

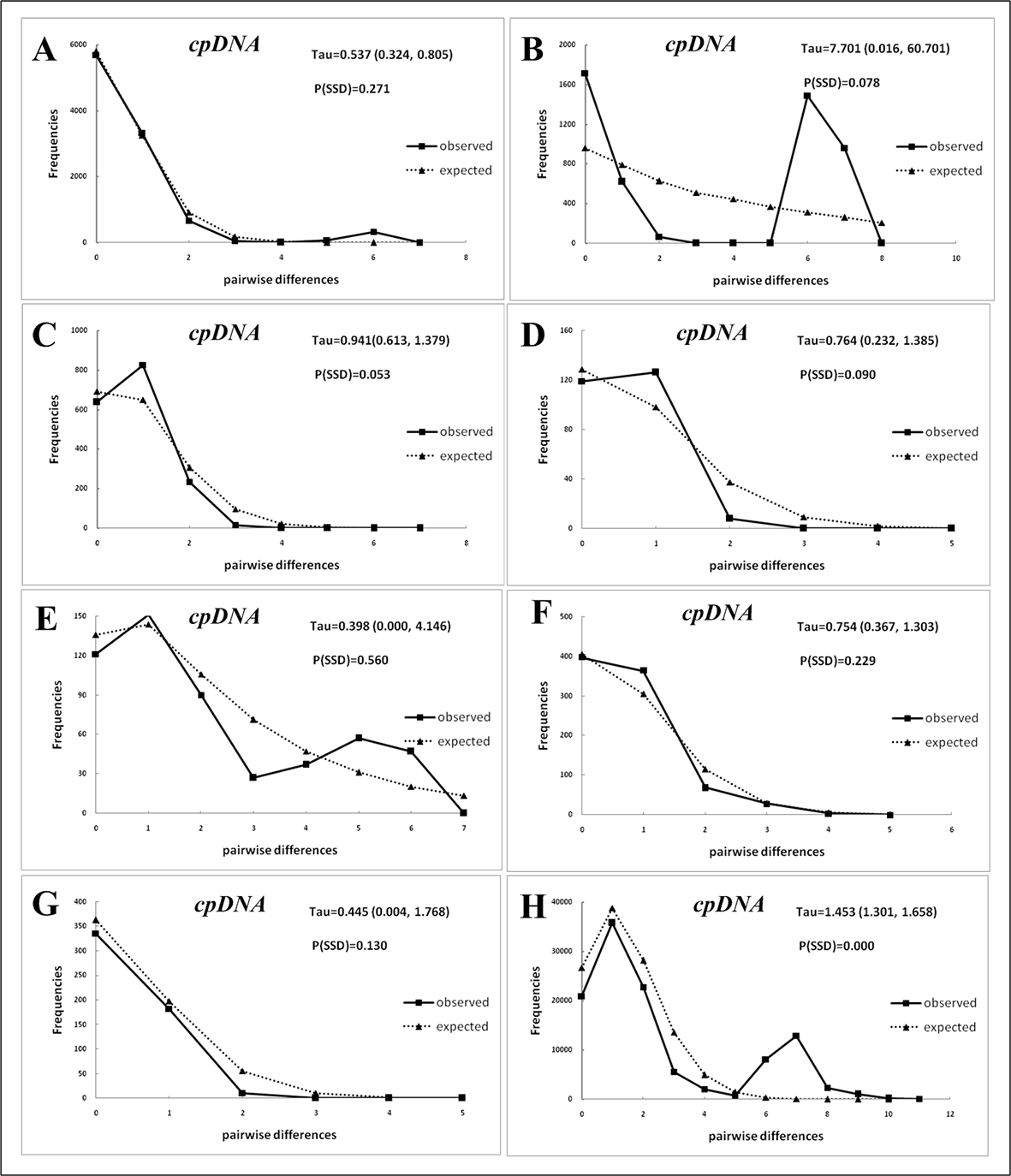

Supplement: Figure S2 — The mismatch distributions for seven fir species in subtropical and temperate China based on cpDNA. A. chensiensis, A. delavayi, A. fabri, A. forrestii, A. georgei, A. recurvata, A. squamata, and the Chinese subtropical and temperate firs as a whole are presented in (A–H), respectively. [file Image2.TIF]

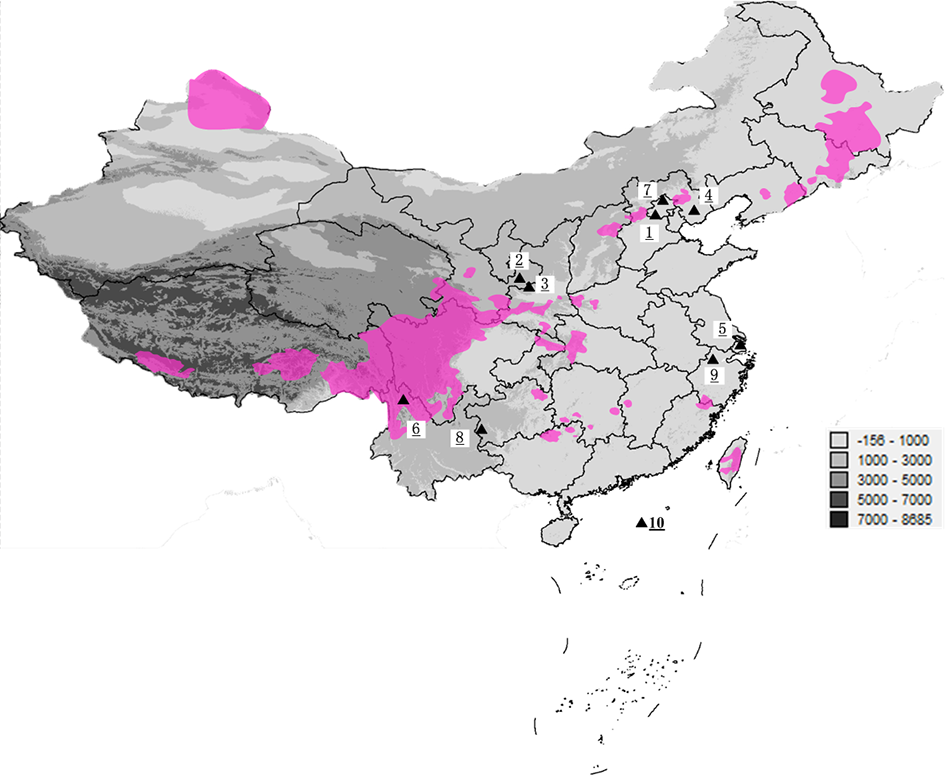

Supplement: Figure S3 — The extant distribution of Abies in China and 10 pollen records during the last glaciation. The red shows the present geographical ranges and the black triangle indicates the distribution of 10 pollen records in the subtropical and temperate China. [file Image3.TIF]

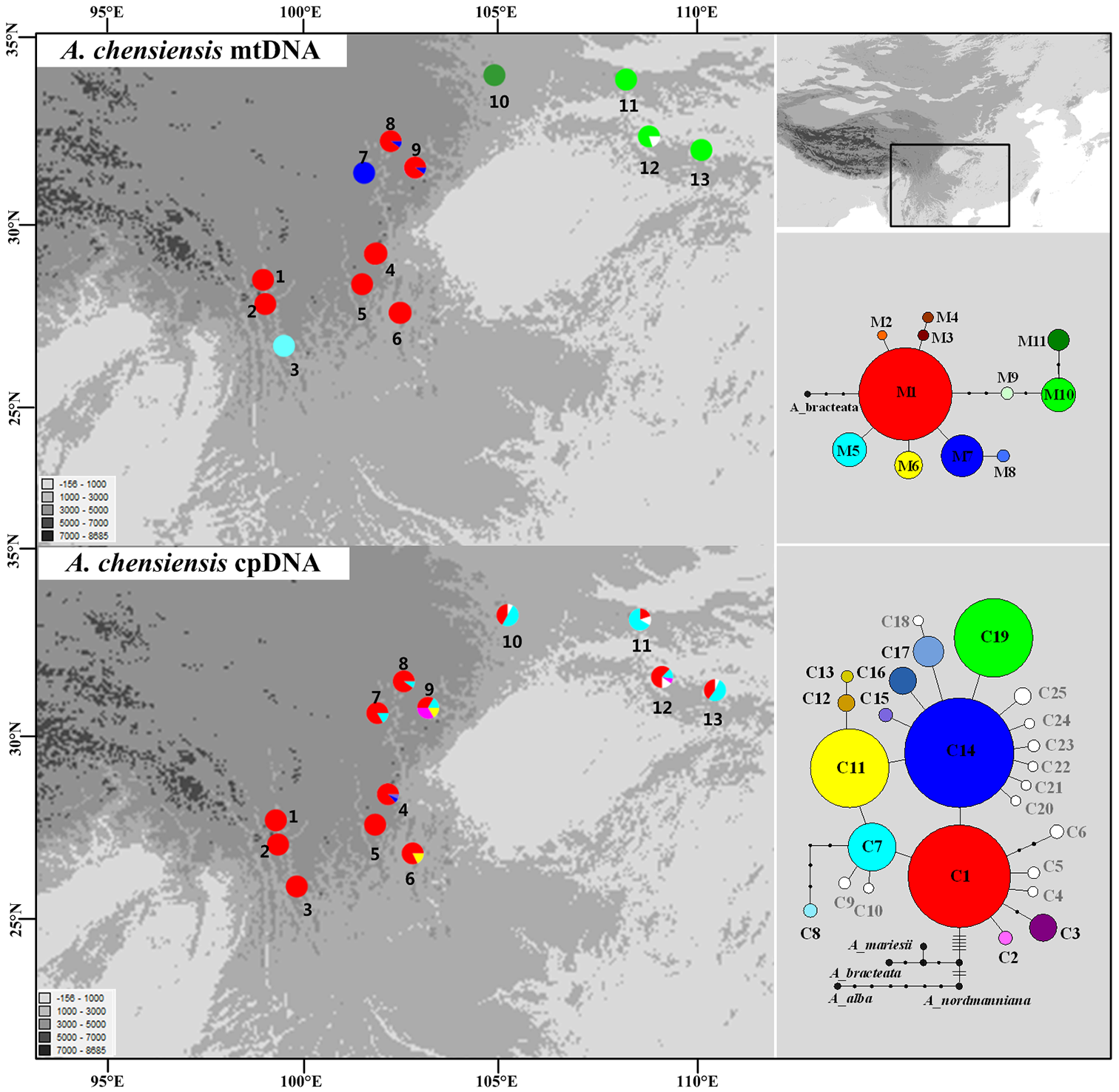

Supplement: Figure S4 — Distributions and networks of mitotypes and chlorotypes recorded in Abies chensiensis. Private chlorotypes are shown in white. See Table S2 for population code numbers. [file Image4.TIF]

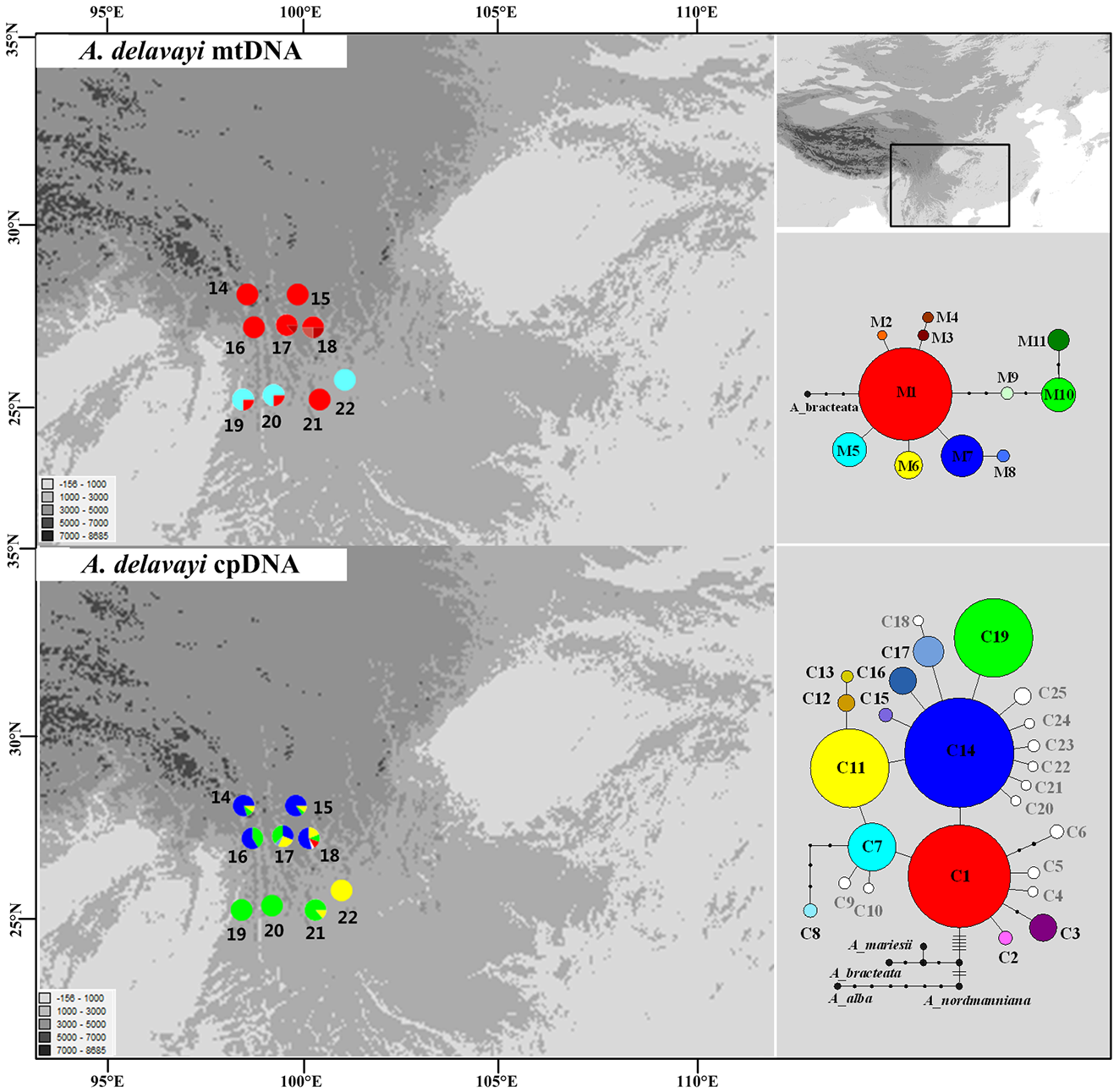

Supplement: Figure S5 — Distributions and networks of mitotypes and chlorotypes recorded in Abies delavayi. Private chlorotypes are shown in white. See Table S2 for population code numbers. [file Image5.TIF]

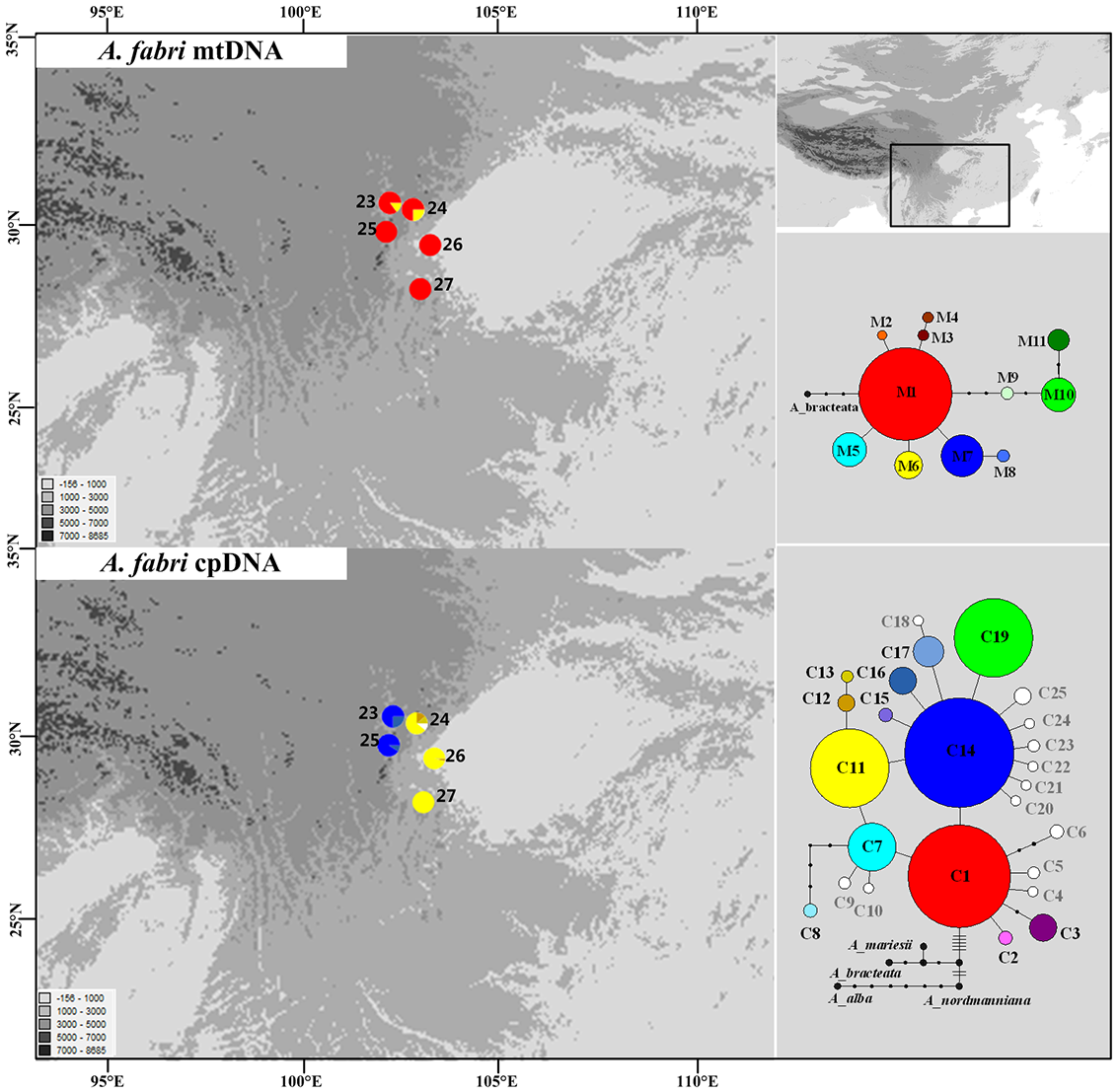

Supplement: Figure S6 — Distributions and networks of mitotypes and chlorotypes recorded in Abies fabri. Private chlorotypes are shown in white. See Table S2 for population code numbers. [file Image6.TIF]
