# Supplementary material for: Elevation Shift in Abies Mill. (Pinaceae) of Subtropical and Temperate China and Vietnam—Corroborative Evidence from Cytoplasmic DNA and Ecological Niche Modeling
Source: Front Plant Sci. 2017 Apr 18;8:578. doi: 10.3389/fpls.2017.00578 (PMC5394127; doi:10.3389/fpls.2017.00578)
Supplement: Table S1 — Ten fossil records of Abies spp. in eastern and central China during the last glaciation. [file Table1.DOC]

**Table S1.** Ten fossil records of *Abies* spp. in eastern and central China during the last glaciation.

| **Code** | **Species** | **Site** | **Latitude**  **(°)** | **Longitude**  **(°)** | **Altitude**  **(m)** | **Archive type** | **Dating method** | **Time span**  **(cal ka BP)** | **References** |
| --- | --- | --- | --- | --- | --- | --- | --- | --- | --- |
| **1** | *Abies* spp. | Fenzhuang | 39.57 | 115.9 | 49 | Fluvial sediment | ^14^C | 15.5–11.5 | Zhang *et al*. 1997 |
| **2** | *Abies* spp. | Suancigou_Feng | 35.51 | 105.81 | 1840 | Loess profile | ^14^C | 22–11.5 | Feng *et al*. 2007 |
| **3** | *Abies* spp. | Suancigou_Tang | 35.5 | 105.83 | 1850 | Loess profile | ^14^C | 22–13.5 | Tang *et al*. 2007 |
| **4** | *Abies* spp. | Yangjiapo | 40.02 | 118.68 | 70 | Fluvial sediment | ^14^C | 22–15.5 | Xu *et al*. 2002 |
| **5** | *Abies* spp. | Shanghai | 31.22 | 121.4 | 4 | Paleo-soil | ^14^C | 22–17 | Wu *et al*. 2002 |
| **6** | *Abies* spp. | Shudu Lake | 27.91 | 99.95 | 3630 | Lake sediment | ^14^C | 22-11 | Cook *et al*. 2011 |
| **7** | *Abies* spp. | Lexinju and Jishuitan,  Beijing | 40.5 | 116.6 | 44 | Paleo-soil | ^14^C | 30-10 | Zhou *et al*. 1978 |
| **8** | *Abies* spp. | Panxian, Guizhou Province | 25.7 | 104.5 | 2050 | Paleo-soil | ^14^C | 30-10 | Zhou *et al*. 1978 |
| **9** | *Abies* spp. | Tianmu Mountain | 30.7 | 119.7 | 450 | Paleo-soil | ^14^C | 30 | Liu and Ye 1977 |
| **10** | *Abies* spp. | northern slope of the South China Sea | 20.1 | 117.38 | -1727 | Coast sediment | ^14^C | 37-10 | Sun and Li 1999 |
