# Supplementary material for: Elevation Shift in Abies Mill. (Pinaceae) of Subtropical and Temperate China and Vietnam—Corroborative Evidence from Cytoplasmic DNA and Ecological Niche Modeling
Source: Front Plant Sci. 2017 Apr 18;8:578. doi: 10.3389/fpls.2017.00578 (PMC5394127; doi:10.3389/fpls.2017.00578)
Supplement: Table S2 — Details of sample locations and sample sizes of 43 populations and outgroups. For each population, estimates of haplotype diversity (h), nucleotide diversity (n) and frequencies of haplotypes for mitochondrial DNA and chloroplast DNA sequences are also indicated.YN, Yunnan; SC, Sichuan; GS, Gansu; SX, Shanxi; CQ, Chongqing; HB, Hubei; GZ, Guizhou; HN, Hunan; GX, Guangxi; N, samples size. *, highly endangered species. #, outgroups. [file Table2.DOC]

**Table S2.** Details of sample locations and sample sizes of 43 populations and outgroups. For each population, estimates of haplotype diversity (*h*), nucleotide diversity (π) and frequencies of haplotypes for mitochondrial DNA and chloroplast DNA sequences are also indicated.

|  |  |  |  |  |  |  | **mtDNA** | | |  | **cpDNA** | | |
| --- | --- | --- | --- | --- | --- | --- | --- | --- | --- | --- | --- | --- | --- |
| **Species** | **Code** | **Location** | **Lat.**  **(N)** | **Long.**  **(E)** | **Alt.**  **(m)** | **N** | ***h*** | ***π*×10^-3^** | **Haplotypes**  (no. of individuals) |  | ***h*** | ***π*×10^-3^** | **Haplotypes**  (no. of individuals) |
| ***A. chensiensis*** | **1** | Deqin YN | 28.18 | 98.90 | 3900 | 12 | 0.00 | 0.00 | M1 (12) |  | 0.00 | 0.00 | C1 (12) |
|  | **2** | Xuelongshan YN | 27.15 | 99.25 | 3400 | 7 | 0.00 | 0.00 | M1 (7) |  | 0.00 | 0.00 | C1 (7) |
|  | **3** | Lanping YN | 26.48 | 99.33 | 3100 | 11 | 0.00 | 0.00 | M5 (11) |  | 0.00 | 0.00 | C1 (11) |
|  | **4** | Jilong SC | 29.03 | 103.25 | 3100 | 12 | 0.00 | 0.00 | M1 (12) |  | 0.32 | 0.69 | C1 (10), C14 (1), C15 (1) |
|  | **5** | Muli SC | 28.07 | 101.17 | 3300 | 11 | 0.00 | 0.00 | M1 (11) |  | 0.00 | 0.00 | C1 (11) |
|  | **6** | Dechang SC | 27.14 | 102.25 | 3000 | 12 | 0.00 | 0.00 | M1 (12) |  | 0.30 | 0.89 | C1 (10), C11 (2) |
|  | **7** | Daofu SC | 30.37 | 101.44 | 2750 | 12 | 0.00 | 0.00 | M7 (12) |  | 0.30 | 0.44 | C1 (10), C7 (2) |
|  | **8** | Lixian SC | 31.37 | 102.49 | 2650 | 12 | 0.17 | 0.22 | M1 (11), M7 (1) |  | 0.30 | 0.44 | C1 (10), C7 (2) |
|  | **9** | Wenchuan SC | 30.55 | 103.03 | 2450 | 8 | 0.25 | 0.32 | M1 (7), M7 (1) |  | 0.82 | 1.78 | C1 (3), C2 (2), C7 (2), C11 (1) |
|  | **10** | Zhouqu GS | 33.33 | 104.2 | 2100 | 11 | 0.00 | 0.00 | M11 (11) |  | 0.64 | 1.06 | C1 (5), C7 (5), C8 (1) |
|  | **11** | Ningshan SX | 33.5 | 108.5 | 2300 | 12 | 0.00 | 0.00 | M10 (12) |  | 0.62 | 1.04 | C1 (3), C7 (7), C9 (2) |
|  | **12** | Dabashan CQ | 31.82 | 109.01 | 2100 | 12 | 0.30 | 0.68 | M9 (2), M10 (10) |  | 0.65 | 1.39 | C1 (7), C2 (1), C7 (2), C8 (2) |
|  | **13** | Shennongjia HB | 31.75 | 110.67 | 2000 | 12 | 0.00 | 0.00 | M10 (12) |  | 0.62 | 1.04 | C1 (5), C4 (1), C7 (6) |
| ***A. delavayi*** | **14** | Gongshan YN | 28.00 | 98.60 | 3300 | 8 | 0.00 | 0.00 | M1 (8) |  | 0.46 | 0.73 | C11 (1), C14 (6), C19 (1) |
|  | **15** | Zhongdian YN | 27.79 | 99.63 | 3800 | 12 | 0.00 | 0.00 | M1 (12) |  | 0.44 | 0.69 | C11 (2), C14 (9), C19 (1) |
|  | **16** | Fugong YN | 27.15 | 98.61 | 3400 | 12 | 0.00 | 0.00 | M1 (12) |  | 0.53 | 0.78 | C14 (7), C19 (5) |
|  | **17** | Weixi YN | 27.14 | 99.39 | 3100 | 12 | 0.17 | 0.19 | M1 (11), M3 (1) |  | 0.74 | 1.47 | C11 (2), C14 (4), C17 (1), C19 (5) |
|  | **18** | Lijiang YN | 27.04 | 100.05 | 3400 | 12 | 0.68 | 1.23 | M1 (6), M3 (3), M4 (3) |  | 0.80 | 1.58 | C1 (2), C11 (2), C14 (5), C19 (2), C22 (1) |
|  | **19** | Lushui YN | 25.58 | 98.41 | 3000 | 12 | 0.41 | 0.53 | M1 (3), M5 (9) |  | 0.00 | 0.00 | C19 (12) |
|  | **20** | Yunlong YN | 25.65 | 99.12 | 3000 | 12 | 0.41 | 0.53 | M1 (3), M5 (9) |  | 0.00 | 0.00 | C19 (12) |
|  | **21** | Dali YN | 25.62 | 100.13 | 3400 | 12 | 0.00 | 0.00 | M1 (12) |  | 0.30 | 0.89 | C11 (2), C19 (10) |
|  | **22** | Binchuan YN | 25.96 | 100.41 | 3100 | 7 | 0.00 | 0.00 | M5 (7) |  | 0.00 | 0.00 | C11 (7) |
| ***A. fabri*** | **23** | Erlangshan SC | 30.07 | 102.30 | 2900 | 12 | 0.33 | 0.37 | M1 (10), M6 (2) |  | 0.41 | 0.60 | C14 (9), C16 (3) |
|  | **24** | Tianquan SC | 30.06 | 102.75 | 2600 | 12 | 0.41 | 0.53 | M1 (9), M7 (3) |  | 0.64 | 1.27 | C11 (7), C12 (3), C13 (1), C23 (1) |
|  | **25** | Kangding SC | 29.55 | 101.53 | 3240 | 12 | 0.00 | 0.00 | M1 (12) |  | 0.17 | 0.24 | C14 (11), C16 (1) |
|  | **26** | Emei SC | 29.51 | 103.34 | 2400 | 12 | 0.00 | 0.00 | M1 (12) |  | 0.17 | 0.24 | C11 (11), C12 (1) |
|  | **27** | Meigu SC | 28.40 | 103.08 | 2600 | 12 | 0.17 | 0.22 | M1 (11), M2 (1) |  | 0.00 | 0.00 | C11 (12) |
| ***A. forrestii*** | **28** | Gongga SC | 29.29 | 100.43 | 3900 | 12 | 0.49 | 0.54 | M1 (8), M6 (4) |  | 0.53 | 0.77 | C14 (7), C16 (5) |
|  | **29** | Jiulong SC | 29.10 | 101.25 | 3600 | 12 | 0.55 | 0.78 | M1 (8), M2 (2), M6 (2) |  | 0.64 | 1.09 | C11 (1), C14 (7), C16 (3), C19 (1) |
| ***A. georgei*** | **30** | Linzhi Tibet | 29.49 | 94.44 | 3900 | 12 | 0.53 | 0.78 | M7 (7), M8 (5) |  | 0.41 | 1.79 | C1 (9), C6 (3) |
|  | **31** | Chaya Tibet | 29.17 | 97.19 | 3800 | 12 | 0.00 | 0.00 | M7 (12) |  | 0.74 | 1.94 | C13 (1), C14 (4), C17 (5), C25 (2) |
|  | **32** | Chaya Tibet | 28.59 | 98.06 | 4000 | 11 | 0.00 | 0.00 | M7 (11) |  | 0.64 | 1.06 | C14 (5), C17 (5), C18 (1) |
| ***A. recurvata*** | **33** | Xinlong SC | 31.20 | 100.16 | 3100 | 11 | 0.00 | 0.00 | M7 (11) |  | 0.18 | 0.27 | C14 (10), C15 (1) |
|  | **34** | Luhuo SC | 31.12 | 100.51 | 3200 | 12 | 0.00 | 0.00 | M7 (12) |  | 0.49 | 0.71 | C11 (4), C14 (8) |
|  | **35** | Litang SC | 30.37 | 100.28 | 3150 | 12 | 0.00 | 0.00 | M7 (12) |  | 0.46 | 1.18 | C10 (1), C14 (9), C15 (1), C24 (1) |
|  | **36** | Yangjiang SC | 30.02 | 100.93 | 2900 | 7 | 0.29 | 0.37 | M1 (1), M7 (6) |  | 0.52 | 0.84 | C1 (5), C14 (1), C15 (1) |
| ***A. squamata*** | **37** | Deqin YN | 28.38 | 99.00 | 4000 | 12 | 0.00 | 0.00 | M1 (12) |  | 0.17 | 0.24 | C14 (11), C19 (1) |
|  | **38** | Batang SC | 29.12 | 99.33 | 4078 | 12 | 0.00 | 0.00 | M1 (12) |  | 0.67 | 1.18 | C14 (7), C16 (2), C17 (1), C19 (1), C21 (1) |
|  | **39** | Xiangcheng SC | 28.34 | 99.84 | 4361 | 12 | 0.00 | 0.00 | M1 (12) |  | 0.53 | 0.85 | C14 (8), C16 (3), C20 (1) |
| ****A. fanjingshanensis*** | **40** | Fanjingshan GZ | 26.88 | 108.77 | 2328 | 11 | 0.00 | 0.00 | M1 (11) |  | 0.69 | 1.88 | C1 (5), C3 (4), C7 (2) |
| ****A. yuanbaoshanensis*** | **41** | Yuanbaoshan GX | 25.29 | 114.38 | 2015 | 12 | 0.00 | 0.00 | M1 (12) |  | 0.17 | 0.49 | C1 (1), C3 (11) |
| ****A. ziyuanensis*** | **42** | Shunhuangshan HN | 26.37 | 111.02 | 1717 | 12 | 0.00 | 0.00 | M1 (12) |  | 0.56 | 1.13 | C1 (8), C5 (2), C7 (1), C11 (1) |
| ****A. fansipanensis*** | **43** | Fansipan Vietnam | 22.40 | 103.90 | 2100 | 4 | 0.00 | 0.00 | M1 (4) |  | 0.00 | 0.00 | C19 (4) |
| **In total** |  |  |  |  | 479 | | 0.61 | 1.59 |  |  | 0.82 | 2.07 |  |
| **^#^A. bracteata** |  | U.K. (cultivated), Royal Botanic Garden Edinburgh | | | | | |  |  |  |  |  |  |
| **^#^A. alba** |  | U.K. (cultivated), Royal Botanic Garden Edinburgh | | | | | |  |  |  |  |  |  |
| **^#^A. nordmanniana** |  | U.K. (cultivated), Royal Botanic Garden Edinburgh | | | | | |  |  |  |  |  |  |
| **^#^A. mariesii** |  | Honshu, Japan |  |  |  | |  |  |  |  |  |  |  |

Abbrevations: YN, Yunnan; SC, Sichuan; GS, Gansu; SX, Shanxi; CQ, Chongqing; HB, Hubei; GZ, Guizhou; HN, Hunan; GX, Guangxi; N, samples size. *：highly endangered species. ^#^：outgroups.

.
