# Supplementary material for: Elevation Shift in Abies Mill. (Pinaceae) of Subtropical and Temperate China and Vietnam—Corroborative Evidence from Cytoplasmic DNA and Ecological Niche Modeling
Source: Front Plant Sci. 2017 Apr 18;8:578. doi: 10.3389/fpls.2017.00578 (PMC5394127; doi:10.3389/fpls.2017.00578)
Supplement: Table S3 — Variable sites of the 11 mitotypes detected in Chinese subalpine and temperate firs. [file Table3.DOC]

| **Mitotype** | Nucleotide variation positions | | | | | | | | | | | |
| --- | --- | --- | --- | --- | --- | --- | --- | --- | --- | --- | --- | --- |
|  | *nad1-2* | | | | | | | *nad5-4* | | | | |
|  |  | 1 | 2 | 2 | 3 | 3 | 3 | | 3 | 7 | 7 | 8 |
|  | 4 | 5 | 0 | 8 | 3 | 4 | 7 | | 8 | 6 | 9 | 0 |
|  | 6 | 1 | 8 | 7 | 8 | 4 | 4 | | 8 | 0 | 2 | 6 |
| M1 | C | G | C | G | G | C | G | | T | ---- | AT | -- |
| M2 | C | G | C | G | G | C | T | | T | ---- | AT | -- |
| M3 | C | - | C | G | G | C | G | | T | ---- | AT | -- |
| M4 | C | - | C | C | G | C | G | | T | ---- | AT | -- |
| M5 | C | G | C | G | G | C | G | | G | ---- | AT | -- |
| M6 | C | G | A | G | G | C | G | | T | ---- | AT | -- |
| M7 | A | G | C | G | G | C | G | | T | ---- | AT | -- |
| M8 | A | G | C | G | G | C | G | | T | CTAT | AT | -- |
| M9 | C | G | C | G | G | C | G | | T | CTAT | -- | AT |
| M10 | C | G | C | G | T | T | G | | T | CTAT | -- | AT |
| M11 | C | G | C | G | T | T | G | | T | ---- | -- | AT |

**Table S3.** Variable sites of the 11 mitotypes detected in Chinese subalpine and temperate

firs*.*
