# Supplementary material for: Elevation Shift in Abies Mill. (Pinaceae) of Subtropical and Temperate China and Vietnam—Corroborative Evidence from Cytoplasmic DNA and Ecological Niche Modeling
Source: Front Plant Sci. 2017 Apr 18;8:578. doi: 10.3389/fpls.2017.00578 (PMC5394127; doi:10.3389/fpls.2017.00578)
Supplement: Table S4 — Variable sites of the 25 chlorotypes detected in Chinese subalpine and temperate firs. #, AAGA; *, ACGGA; &, TATCTA; α, ATTATTG; β, CTATTGTAAAGATATTAGTTGCTCAAGG; γ, ACTAC. [file Table4.DOC]

**Table S4.** Variable sites of the 25 chlorotypes detected in Chinese subalpine and temperate firs*.*

| **Chlorotype** | Nucleotide variation positions | | | | | | | | | | | | | | | | | | | | | | |
| --- | --- | --- | --- | --- | --- | --- | --- | --- | --- | --- | --- | --- | --- | --- | --- | --- | --- | --- | --- | --- | --- | --- | --- |
|  | *trnS-trnG* | | | | | | | | | | | | | | | | | | | | | | |
|  |  |  | 1 | 1 | 1 | 2 | 3 | 3 | 3 | 3 | 3 | 3 | 4 | 4 | 4 | 4 | 5 | 5 | 5 | 6 | 6 | 6 | 7 |
|  | 7 | 9 | 1 | 8 | 9 | 6 | 1 | 2 | 4 | 5 | 6 | 6 | 1 | 4 | 5 | 5 | 2 | 5 | 5 | 2 | 5 | 6 | 1 |
|  | 1 | 4 | 5 | 0 | 5 | 6 | 9 | 1 | 0 | 4 | 1 | 5 | 1 | 3 | 4 | 8 | 9 | 0 | 9 | 0 | 1 | 3 | 5 |
| C1 | - | T | ACC | T | A | * | G | A | & | α | T | G | - | A | C | γ | C | G | G | G | C | A | C |
| C2 | - | T | ACC | T | A | * | G | T | & | α | T | G | - | A | C | γ | C | G | G | G | C | A | C |
| C3 | - | T | ACC | G | A | * | G | A | & | α | T | G | - | A | C | γ | C | G | G | G | T | A | C |
| C4 | - | T | ACC | T | A | * | G | A | & | α | T | G | - | A | T | γ | C | G | G | G | C | A | C |
| C5 | - | T | ACC | T | A | * | A | A | & | - | T | G | - | A | C | γ | C | G | G | G | C | A | C |
| C6 | - | T | TAA | T | A | * | G | A | & | α | T | G | - | A | C | γ | C | G | G | G | C | A | C |
| C7 | - | T | ACC | T | A | * | G | A | & | α | T | G | - | A | C | γ | C | G | G | A | C | A | C |
| C8 | - | T | ACC | T | A | * | G | A | & | α | T | G | - | A | C | - | C | G | G | A | C | A | C |
| C9 | - | C | ACC | T | A | * | G | A | & | α | T | G | - | A | C | γ | C | G | G | A | C | A | C |
| C10 | - | T | ACC | T | A | * | G | A | & | α | T | G | - | A | C | γ | C | G | G | A | C | T | C |
| C11 | - | T | ACC | T | A | * | G | A | & | α | G | G | - | A | C | γ | C | G | G | A | C | A | C |
| C12 | - | T | ACC | T | A | * | G | A | & | α | G | A | - | A | C | γ | C | G | G | A | C | A | C |
| C13 | - | T | ACC | T | A | * | G | A | & | α | G | A | β | A | C | γ | C | G | G | A | C | A | C |
| C14 | - | T | ACC | T | A | * | G | A | & | α | G | G | - | A | C | γ | C | G | G | G | C | A | C |
| C15 | - | T | ACC | T | G | * | G | A | & | α | G | G | - | A | C | γ | C | G | G | G | C | A | C |
| C16 | - | T | TAA | T | A | * | G | A | & | α | G | G | - | A | C | γ | C | A | G | G | C | A | C |
| C17 | - | T | ACC | T | A | * | G | A | & | α | G | G | - | A | C | γ | G | G | G | G | C | A | C |
| C18 | # | T | ACC | T | A | * | G | A | & | α | G | G | - | A | C | γ | G | G | G | G | C | A | C |
| C19 | - | T | ACC | T | A | * | G | A | - | α | G | G | - | A | C | γ | C | G | G | G | C | A | C |
| C20 | - | T | ACC | T | A | * | G | A | & | α | G | G | - | A | C | γ | C | G | A | G | C | A | C |
| C21 | - | T | ACC | T | A | * | G | A | & | α | G | G | β | T | C | γ | C | G | G | G | C | A | C |
| C22 | - | T | ACC | T | A | * | G | A | & | α | G | G | - | A | C | γ | C | G | G | G | C | A | T |
| C23 | - | T | ACC | T | A | * | G | A | & | α | G | A | - | A | C | γ | C | G | G | G | C | A | C |
| C24 | - | T | ACC | T | A | * | G | A | & | α | G | G | - | A | C | γ | C | G | G | G | C | T | C |
| C25 | - | T | ACC | T | A | - | G | A | & | α | G | G | - | A | C | γ | C | G | G | G | C | A | C |

#=AAGA; *=ACGGA; &=TATCTA; α=ATTATTG; β=CTATTGTAAAGATATTAGTTGCTCAAGG; γ=ACTAC.
