# Supplementary material for: Elevation Shift in Abies Mill. (Pinaceae) of Subtropical and Temperate China and Vietnam—Corroborative Evidence from Cytoplasmic DNA and Ecological Niche Modeling
Source: Front Plant Sci. 2017 Apr 18;8:578. doi: 10.3389/fpls.2017.00578 (PMC5394127; doi:10.3389/fpls.2017.00578)
Supplement: Table S5 — Species names and GenBank accession numbers of DNA sequences used in this study. * means newly generated sequences. [file Table5.DOC]

**Table S5.** Species names and GenBank accession numbers of DNA sequences used in this study. *: means newly generated sequences.

| **Species** | **GenBank accession number** | | |
| --- | --- | --- | --- |
|  | ***nad1-2*** | ***nad5-4*** | **trnS-G** |
| ***A. chensiensis*** | KJ146795  KJ146796  KJ146797  KJ146798  KJ146799  KJ146800 | KJ146805  KJ146806 | KJ146813  KJ146814  KJ146815  KJ146816  KJ146817  KJ146818  KJ146819  KJ146820 |
| ***A. delavayi*** | KP635976*  KP635977*  KP635978*  KP635979* | KP635993* | KP636003*  KP636004*  KP636005*  KP636006*  KP636007*  KP636008* |
| ***A. fabri*** | KP635980*  KP635981*  KP635982*  KP635983* | KP635994* | KP636009*  KP636010*  KP636011*  KP636012*  KP636013*  KP636014* |
| ***A. forrestii*** | KP635984*  KP635985*  KP635986* | KP635995* | KP636015*  KP636016*  KP636017*  KP636018* |
| ***A. georgei*** | KP635987* | KP635996*  KP635997* | KP636019*  KP636020*  KP636021*  KP636022*  KP636023*  KP636024*  KP636025* |
| ***A. recurvata*** | KJ146802  KJ146803 | KJ146807 | KJ146827  KJ146828  KJ146829  KJ146830  KJ146831  KJ146832 |
| ***A. squamata*** | KP635988* | KP635998* | KP636026*  KP636027*  KP636028*  KP636029*  KP636030*  KP636031* |
| ***A. fanjingshanensis*** | KP635989* | KP635999* | KP636032*  KP636033*  KP636034* |
| ***A. yuanbaoshanensis*** | KP635990* | KP636000* | KP636035*  KP636036* |
| ***A. ziyuanensis*** | KP635991* | KP636001* | KP636037*  KP636038*  KP636039*  KP636040* |
| ***A. fansipanensis*** | KP635992* | KP636002* | KP636041* |
| ***A. bracteata*** | KJ146794 | KJ146804 | KJ146810 |
| ***A. alba*** |  |  | KJ146809 |
| ***A. nordmanniana*** |  |  | KJ146812 |
| ***A. mariesii*** |  |  | KJ146811 |
